# Determinants of establishment success: Comparing alien and native freshwater fishes in Taiwan

**DOI:** 10.1371/journal.pone.0236427

**Published:** 2020-07-23

**Authors:** Shih-Hsiung Liang, Bruno Andreas Walther, Bao-Sen Shieh

**Affiliations:** 1 Department of Biotechnology, National Kaohsiung Normal University, Kaohsiung, Taiwan; 2 Department of Biological Sciences, National Sun Yat-sen University, Kaohsiung, Taiwan; 3 Department of Biomedical Science and Environmental Biology, Kaohsiung Medical University, Kaohsiung, Taiwan; 4 Department of Medical Research, Kaohsiung Medical University Hospital, Kaohsiung, Taiwan; Universidad de la Republica Uruguay, URUGUAY

## Abstract

Many parts of Asia, including Taiwan, have suffered severely from freshwater fish invasions. However, few studies using an assemblage approach have been conducted in the region so far that would help to prioritize suitable preventive actions. In this study, we focused on the invasion process from the import stage to the establishment stage, and defined establishment success as the success during this predefined process. We used datasets of freshwater fish assemblages in Taiwan to (1) compare established versus non-established alien species to distinguish the determinants of establishment success, and (2) to use these determinants to test a life history hypothesis which predicts that the magnitudes of the determinants should be significantly different between established alien species and native species. We collated a dataset for freshwater fish species which were imported into Taiwan (n = 118) of which some successfully established (n = 26), and another dataset for freshwater fish species native to Taiwan (n = 77). For each imported species, we collected data for 17 variables, including two phylogenetic, two human-use, two invasion history, and 11 life history variables. We then used decision tree methods, which have advantages in analyzing datasets with many variables of mixed types without the need to make assumptions about data distributions and input data for missing values. Our results showed that aquaculture use and maximum body length were the most important determinants for predicting establishment success of alien freshwater fish in Taiwan. Comparing five important determinants between established alien versus native species showed that the established alien species were significantly more often used in aquaculture, were associated with a higher number of established countries, and had a larger body length and greater highest water temperature tolerance than the native species. We thus conclude that our results provided evidence to support our stated hypothesis. We suggest that aquaculture use, measures of body size, and the number of previously invaded countries may alert researchers and conservation managers to species with a high establishment potential, especially for countries with similar conditions as those in Taiwan.

## Introduction

Fishes are one of the most successful invasive taxa in the world [1], partly because they are very popular with people for food, recreational fisheries, and aquarium fishkeeping [2]. According to Gozlan’s [3] global analysis, at least 624 alien fish species had already invaded non-native ranges by 2007. Consequently, fish introductions have changed freshwater fish assemblages worldwide [4] and resulted in the global trend of homogenization of freshwater fish assemblages [5]. In addition to the effects of fish introductions on global biodiversity, various ecological and economic impacts of fish invasions have also been documented at the regional and local level [6, 7].

In local regions, many invasions of freshwater fish have had catastrophic ecological consequences [8], and these consequences are generally irreversible. Therefore, studies of fish invasions in local regions have focused not only on the eradication of invasive species after their establishment but, more importantly, the prevention of their introduction and their establishment [e.g., 9, 10]. In order to prioritize preventive management actions and policies, it is important to study entire alien fish assemblages because such analyses allow us to distinguish those species-specific traits which are important in establishment success, defined as the establishment of a self-sustaining population [11]. While global analyses consider a very large number of species, two important analytical considerations arise when we attempt to study local fish assemblages: (1) the use of different comparisons (e.g., native versus alien, introduced versus established) in order to investigate specific invasion hypotheses more comprehensively as suggested by van Kleunen et al. [12], and (2) the inclusion of many possible determinant factors (or variables) of establishment success while having to deal with a considerably smaller sample size of fish species.

The second consideration arises because sample sizes of the included study species are invariably smaller at local scales than at the global scale, while the number of factors which determine establishment success remains high. For example, Ricciardi et al. [13] reviewed 19 published hypotheses regarding the ecological impact of alien species and found that species traits, ecosystem traits, community structure, ecological niches, changes in the abiotic environment, organismal influx, and possible synergistic effects between these various factors may all influence whether a species establishes and what kind of ecological impact the establishment has. Furthermore, biological traits which predict invasion success may also vary depending on the investigated invasion stage [14], biological group [15], or geographical region and scale [16, 17]. It is thus important to consider as many factors as possible when trying to explain the invasion success of alien freshwater fishes within local regions.

One possible solution to the conundrum of smaller sample sizes but many factors is the use of decision tree (DT) methods, which are based on the machine learning algorithms. They have three advantages over more traditional parametric methods: (1) they make no assumptions about data distributions; (2) no need to input data for missing values; and (3) they can analyze datasets with many factors of mixed types (nominal, ordinal, and interval variables) [18]. Kolar and Lodge [16] first applied the DT methods to investigate a relatively small data set of 45 fish species for risk assessment of invasion in the Great Lakes. Keller et al. [19] further used the same dataset (45 species and 17 associated factors) to demonstrate that DT methods performed as well as logistic regression and outperformed other methods in accuracy. Assemblage studies of freshwater fish invasion in Asia have also been hampered by the limitation of small sample size and missing data, and few studies using DT methods have been conducted so far. Therefore, we applied the DT methods in studying the freshwater fish assemblages of Taiwan.

Asian regions have suffered severely from freshwater fish invasions [e.g., 20, 21]. Although Taiwan is a relatively small island found in East Asia, it is a subtropical to temperate island with a relatively high level of biodiversity and endemism [22]. However, due to various factors, it has also experienced a relatively high number of alien species invasions (for amphibians [23], reptiles [24], birds [25], and aquatic invertebrates [26]), including freshwater fishes. At least 293 alien fish species were imported into Taiwan as pets (also called ornamental fish) as of 2004, and of these, 16 fish species successfully established [20]. The four sequential stages of the invasion process are: import, introduction, establishment, and spread [11]. An imported alien species first has to be introduced into the wild, but not all introduced species become established species.

In this study, we focused exclusively on the invasion process from import stage to establishment stage because it is impossible in Taiwan to monitor all introduction events and to assess the success of the introduction stage [11]. For example, there is a cultural tradition of releasing captive animals into the wild (private or prayer release) [e.g., 27] which most people keep secret because it is illegal. Therefore, there are no reliable records about the timing, location, and frequency of alien fish introductions into Taiwan’s freshwater habitats. The same is true for escapees from commercial fisheries or the release of pet fishes [20] which almost certainly happen undetected on a regular basis in Taiwan. For the purpose of our present study, we thus identified establishment success from import stage to establishment stage; an established alien species is defined as an imported alien species which had succeeded during the invasion process from import stage to establishment stage in Taiwan, whereas a non-established alien species is defined as an imported alien species which had failed during the predefined process (i.e. it was either not introduced or introduced but failed to establish). Furthermore, we did not consider the spreading stage.

Using datasets of freshwater fish assemblages imported and native to Taiwan, our study objectives were (1) to use DT methods to compare established versus non-established alien species which enabled us to distinguish those variables (i.e. determinants) which explain establishment success, and (2) to use these important determinants to compare established alien species to native species. We used the results of the latter comparison to then investigate a life history hypothesis [12] for establishment success of freshwater fishes, in which we predict that the magnitudes of the determinants (those which were shown to be important for establishment success from our previous results) should be significantly different between established alien species and native species, as suggested by the previous studies [28,29,30].

## Materials and methods

### Comparison of established species vs. non-established alien species

#### Data set

We updated the dataset of Liang et al. [20] by including all available new information up to 2014, and we used this updated dataset. Therefore, we used the list of 293 alien fish species imported to Taiwan published in Liang et al. [31] and originally used in Liang et al.’s [20] analysis. We then excluded all saltwater fish species as well as any freshwater fish species for which we could not collect data for the predictor variables (Table 1). Our dataset was thus reduced to 118 species (S1 Table) imported into Taiwan. We used this dataset for the comparison of established alien species versus non-established alien species.

**Table 1:** Descriptions of variables used in our analyses.

Variable abbreviation	Data type	Description
Predictor variable		
ORDER	Nominal	Order
FAMILY	Nominal	Family of fish species
LOVERLAP	Ordinal	Latitudinal overlap of the native range with Taiwan’s latitudinal range: 0 for no overlap (0%), 1 for partial overlap, 2 for complete overlap (100%)
DIET	Ordinal	1 for detritivore, 2 for herbivore, 3 for omnivore, 4 for carnivore
AQUAC	Ordinal	Aquaculture use (or fish farming): 1 for no, 2 for yes
PET	Ordinal	Aquarium fish keeping in Taiwan: 1 for no, 2 for yes
PHL	Interval	The lowest pH level
PHH	Interval	The highest pH level
DHL	Interval	The lowest water hardness level
DHH	Interval	The highest water hardness level
TEMPL	Interval	The lowest water temperature tolerance
TEMPH	Interval	The highest water temperature tolerance
MAXL	Interval	The maximum body length
INTROD	Interval	The number of countries where the species was non-native but was introduced
ESTAB	Interval	The number of countries where the species was non-native but has since established itself
FOODIT	Interval	The number of food items (the number of food categories such as insects, annelids, crustaceans, etc.)
FECUN	Interval	Fecundity. The maximum number of eggs an animal produces during each reproductive cycle was recorded.
Outcome variable		
SUCCESS	Binary	1 for established species, 0 for non-established species.

In order to document all the establishment records of these 118 fish species up to December 2014, we continuously (1) checked information from Taiwanese websites dedicated to natural history observations of fishes (e.g., https://tesri.tesri.gov.tw), (2) remained in contact with Taiwan’s ichthyologist experts, and (3) checked for and then included any relevant publications [31–36]. Using this information, we established that 26 out of the total of 118 species had successfully established themselves in Taiwan up to December 2014 (S1 Table) which means 10 more species had established themselves since the original analysis published in Liang et al. [20].

For each of the 118 species, we collected 17 variables, including two nominal ones, four ordinal ones, and 11 interval ones (Table 1). Among those 17 variables, two variables are related to phylogenetic traits (ORDER, FAMILY), two concern human use traits (AQUAC, PET), two consider invasion history traits (INTRO, ESTAB), and 11 are related to life history traits (LOVERLAP, DIET, PHL, PHH, DHL, DHH, TEMPL, TEMPH, MAXL, FOODIT, FECUN). We gathered this species information from two databases: Fishbase (www.fishbase.org) and IUCN (www.iucn.org). All the data is available in S1 Table. The data obtained from the databases for each species were collected from various freshwater systems, such as reservoirs, ponds, lakes, streams, or rivers.

#### Variable exploration

To explore the effect of a single variable on the predefined process (from import stage to establishment stage), we compared the differences between established alien species (n = 92) and non-established alien species (n = 26) using Fisher’s exact tests for each ordinal variable and Wilcoxon rank sum tests for each interval variable. In the present study, we did not identify those species which succeeded at the introduction stage but failed at the establishment stage from the 92 non-established species (see reasoning in Introduction), Therefore, the effect of each variable on the success of the establishment stage was explored by using a random sampling process to simulate a sample of 26 introduced species out of the 92 non-established species. The number of 26 was chosen to match the sample size of the established species. That is, we randomly selected 26 species out of the 92 non-established alien species, and compared these 26 randomly selected species with the 26 established species using Fisher’s exact tests for each ordinal variable and Wilcoxon rank sum tests for each interval variable. The random sampling process and the statistical comparison were repeated 1000 times, and the number of significant results for each variable was evaluated. The significance level was set at 0.05 for all two-sided tests. We used JMP Pro 14.2.0 to perform the randomization process and analyses.

#### Establishment success model

We produced the DT models and variable treatments using SAS Enterprise Miner 13.1. To investigate the possible effects of taxon variables (FAMILY and ORDER) on the performance of DT models, we conducted two variable treatments for modeling: (I) including all variables, and (II) excluding these two nominal taxon variables.

To predict establishment success, we generated four DT models (DT_no bagging, DT_bagging 90%, gradient boosting, and HP forest) each for the two variable treatments. DT_no bagging indicates the decision tree modeling process in SAS Enterprise Miner 13.1. which constructs a layered tree model using the traditional classification tree method; DT_bagging 90% indicates the same modeling process as DT_no bagging but with bagging 90% of the dataset 50 times. Gradient boosting indicates the gradient boosting modeling process, which resamples the dataset to produce a series of decision trees in order to build a single predictive model; and HP forest indicates the random forest modeling process, which builds many parallel trees forming a forest [37].

Data partitioning was set to 50% of data for training and 50% of data for validation, with the program randomly partitioning the data but also ensuring that 50% of the successfully established species and 50% of the failed species end up in each of the training and validation data sets (or 13 and 46 species, respectively, in each data set). In addition, because of our small data set, we also performed cross-validation in model training, which is a recommended procedure for the analysis of small data sets [38].

To compare different models, we calculated five performance measures using only the validation data: (1) the area under the receiver operating characteristic curve (AUC), (2) the specificity, (3) the precision, (4) the recall, and (5) the accuracy for each DT model; we then summed up the five values to choose the model with the highest total score as our final model following the procedure used in Liang et al. [25].

**Fig 1:**
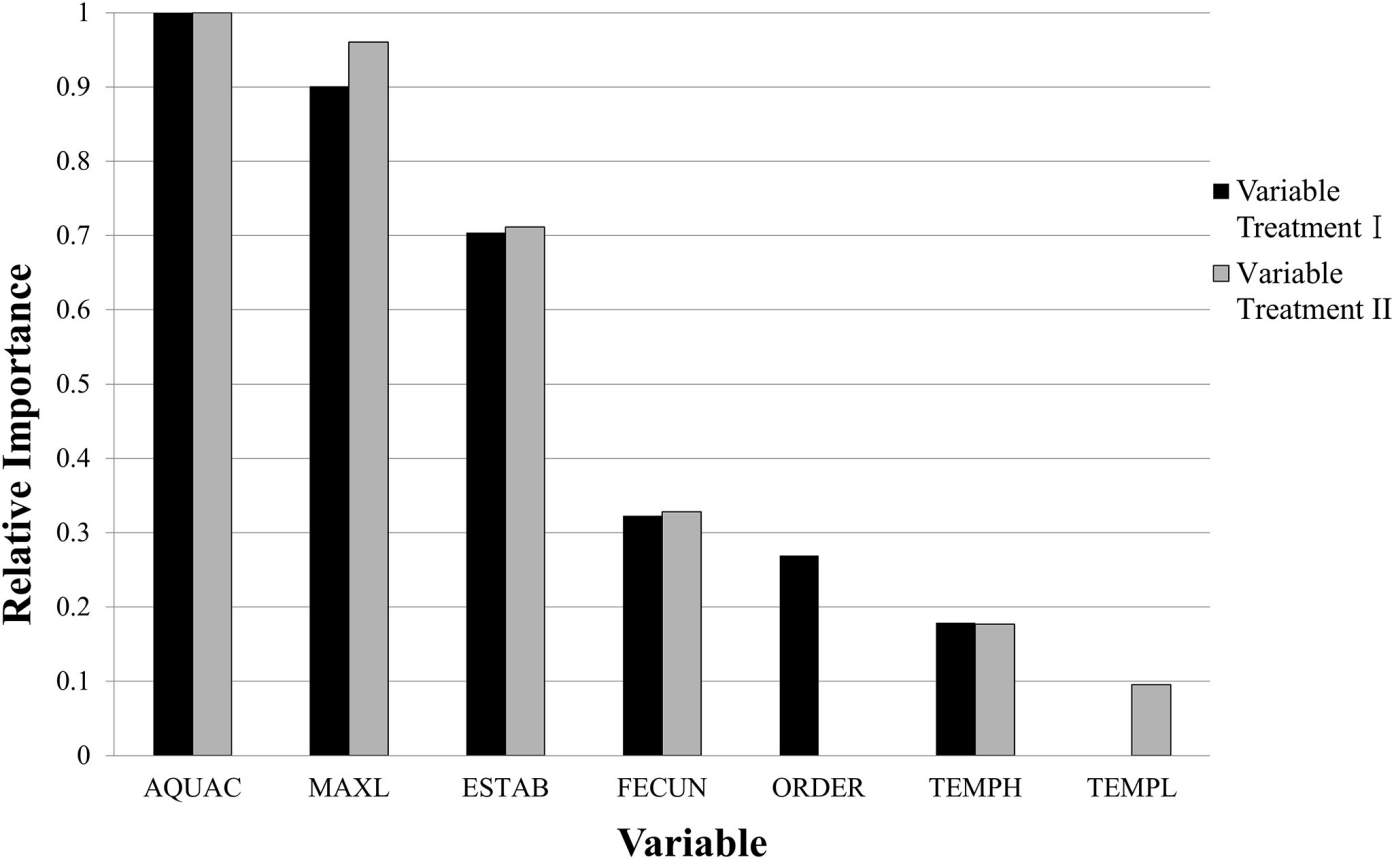
Relative importance of variables in the establishment success model based only on validation data using the gradient boosting approach (black bars for variable treatment I, and grey bars for variable treatment II) (see S1 Table for species list and associated variable information).

**Fig 2:**
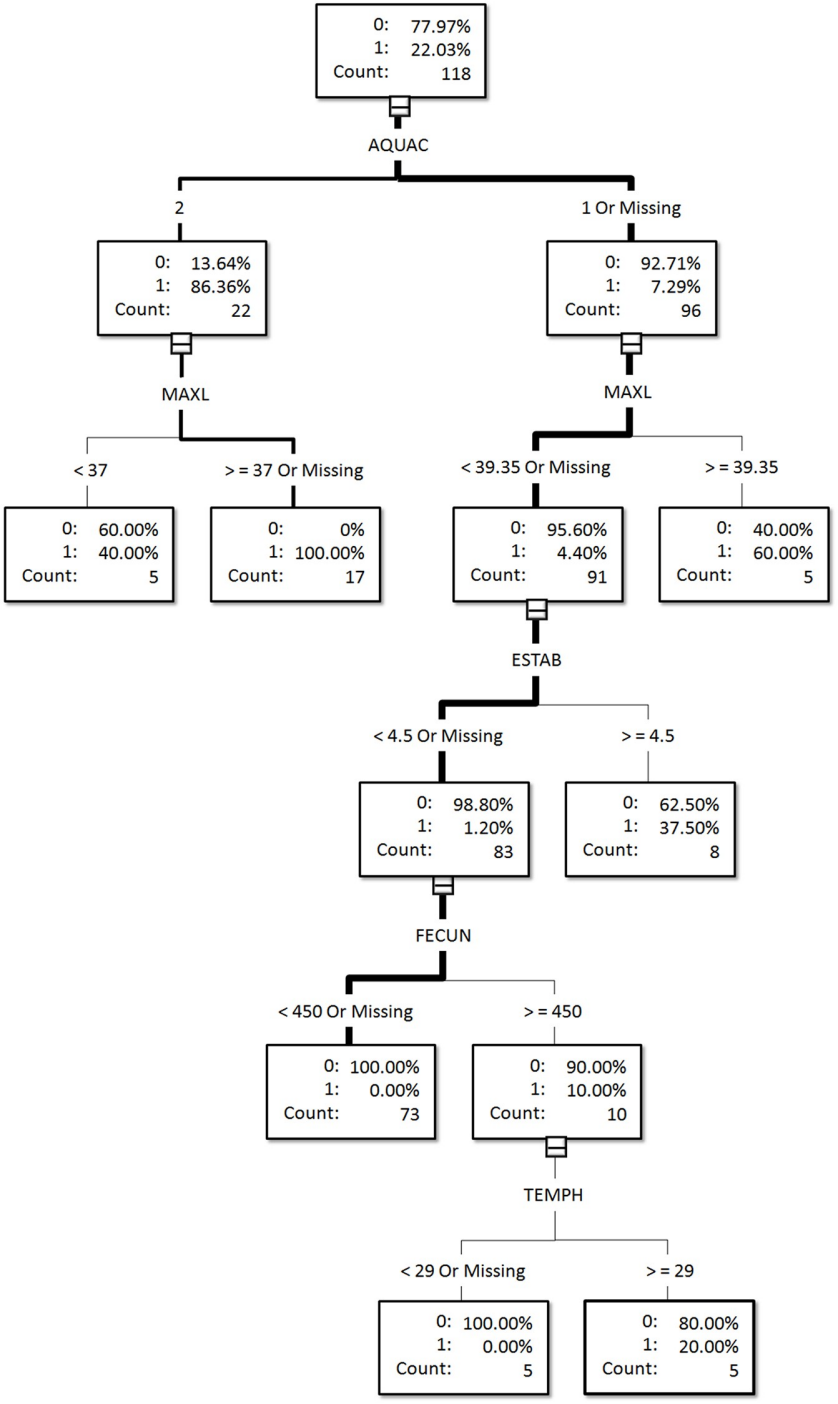
The visual output of the establishment success model based on the interactive classification tree generated by choosing the five most important variables of the gradient boosting approach (118 species of which 26 established; see S1 Table for species list and associated variable information).

The final model provided a list of six variables ranked by their relative importance (variable treatment II in Fig 1). We then used these top-ranked variables to illustrate the conditional probabilities of successful and failed establishments (Fig 2 which was generated by the interactive DT option in SAS). This option allowed us to choose the order of the most important variables in the DT, and the interactive DT option resulted in only five variables being retained (see Fig 2).

The same five top-ranked variables were then also used in the following comparison of established alien species versus native species (see below).

### Comparison of established alien species vs. native species

We used the list of native freshwater fish species published by Chen et al. [39] and excluded all brackish or estuary fishes, which resulted in a list of 77 native freshwater fish species (all the data is available in S2 Table). For each of these 77 species, we collected data for the five top-ranked variables (see above) from the fish database of Taiwan (fishdb.sinica.edu.tw) and Fishbase (www.fishbase.org). We then compared these 77 native species to the 26 established alien species (see above).

We performed (1) chi-square tests for ordinal variables and (2) Wilcoxon rank sum tests for interval variables in order to compare established alien species to native species.

## Results

### Comparison of established vs. non-established alien species

The results of our analyses on single variable basis showed that two ordinal variables (LOVERLAP, AQUAC) and five interval variables (MAXL, INTROD, ESTAB, FOODIT, FECUN) were significantly different in both comparison 1 (92 non-established vs. 26 established alien species) and comparison 2 (26 randomly selected non-established vs. 26 established alien species) (Table 2). However, two numerical variables, TEMPL and TEMPH, were significantly different only in comparison 1 but not comparison 2 (Table 2).

**Table 2:** Comparison between non-established and established alien species for numerical variables.

Variable^a^	Comparison 1^b^ (92 non-established vs. 26 established)	Comparison 2 (26 randomly selected non-established vs. 26 established)
LOVERLAP	**(P < .001)	* (983/1000)
DIET	ns (P = 1)	ns (1/1000)
AQUAC	**(P < .001)	** (1000/1000)
PET	ns (P = 0.398)	ns (2/1000)
PHL	ns (P = 0.745)	ns (15/1000)
PHH	ns (P = 0.766)	ns (7/1000)
DHL	ns (P = 0.298)	ns (26/1000)
DHH	ns (P = 0.156)	ns (71/1000)
TEMPL	**(P = 0.002)	ns (949/1000)
TEMPH	*(P = 0.032)	ns (338/1000)
MAXL	**(P < .001)	** (1000/1000)
INTROD	**(P < .001)	** (1000/1000)
ESTAB	**(P < .001)	** (1000/1000)
FOODIT	**(P < .001)	* (983/1000)
FECUN	**(P < .001)	** (1000/1000)

^a^: See Table 1 for descriptions of variables.

^b^: Wilcoxon rank sum tests for interval variables and Fisher’s exact tests for ordinal variables; * for significance level of 0.05 (two-sided), and ** for significance level of 0.01 (two-sided).

^c^: * for more than 950 significant results in 1000 comparisons, ** for more than 990 significant results in 1000 comparisons; the significance level for each comparison was set at 0.05 for two-sided tests.

We performed DT methods on the dataset of 26 established alien and 92 non-established alien species with all the 17 variables (S1 Table). Using only the validation data, the gradient boosting model, which is one of the four DT models which we used, had the highest total score (i.e., performed best overall) and also the highest precision, recall, and accuracy across the two variable treatments (Table 3). Therefore, we considered the gradient boosting model as our final DT model and only considered its results from hereupon.

**Table 3:** Comparison of five performance measures among our four establishment success models based on validation data of alien fishes in Taiwan, separately for two variable treatments (see Methods for details).

Variable Treatment I.						
Model	AUC	Specificity	Precision	Recall	Accuracy	Total
DT_no bagging	0.908	0.957	0.818	0.692	0.898	4.273
DT_bagging 90%	0.908	0.957	0.818	0.692	0.898	4.273
Gradient Boosting	0.903	0.978	0.909	0.769	0.932	4.492
HP Forest	0.941	0.978	0.875	0.538	0.881	4.214
Variable Treatment II						
Model	AUC	Specificity	Precision	Recall	Accuracy	Total
DT_no bagging	0.908	0.957	0.818	0.692	0.898	4.273
DT_bagging 90%	0.500	1.000	0/0	0.000	0.780	2.280
Gradient Boosting	0.913	0.978	0.909	0.769	0.932	4.502
HP Forest	0.945	0.978	0.900	0.692	0.915	4.431

Looking across the two different variable treatments, the gradient boosting model performed best with variable treatment II (which excluded the taxon variables) (Table 3). However, the total score only increased by 0.01 (or 0.2%) comparing variable treatment I to variable treatment II. Four out of the five performance measures remained the same across variable treatments. Furthermore, the accuracy values were consistently very high (0.932) whereas the values of recall were relatively low when compared with the other performance measures.

In all the gradient boosting models, aquaculture (AQUAC) and the maximum body length (MAXL) were the two most important variables in predicting the establishment success for the imported alien freshwater fish in Taiwan. Furthermore, their relative importance values using only the validation data were 1.00 and 0.90–0.96, respectively (Fig 1). Besides these two top variables, the variables with relative importance values > 0 were the number of established countries (ESTAB), the fecundity (FECUN), ORDER, the highest water temperature tolerance (TEMPH), and the lowest water temperature tolerance (TEMPL).

To illustrate the conditional probabilities of successful and failed establishment (namely, our establishment success model), we used the five top variables shown in Fig 1 and used in variable treatment II (which achieved the highest total scores, see Table 3) to construct an interactive DT for the entire 118 species (Fig 2). The probability of successful establishment of fish species used in aquaculture (AQUAC code = 2) was 86.36%. The probability of successful establishment increased to 100% if the maximum body length of the species was ≥ 37 cm. Therefore, these two factors alone, namely aquaculture use and maximum body length, were sufficient to predict the successful establishment of alien freshwater fish species in Taiwan.

The probability of failed establishment was 92.71% for fish species without aquaculture use (AQUAC code = 1). However, to increase the probability of failed establishment to 100%, three more conditions had to be met: the maximum body length (MAXL) < 39.35 cm, the number of established countries (ESTAB) < 4.5, and the fecundity (FECUN) < 450 (Fig 2). In other words, every one of these four factors was needed to completely predict the failed establishment of alien freshwater fish species in Taiwan (Fig 2).

### Comparison of established alien species vs. native species

**Fig 3:**
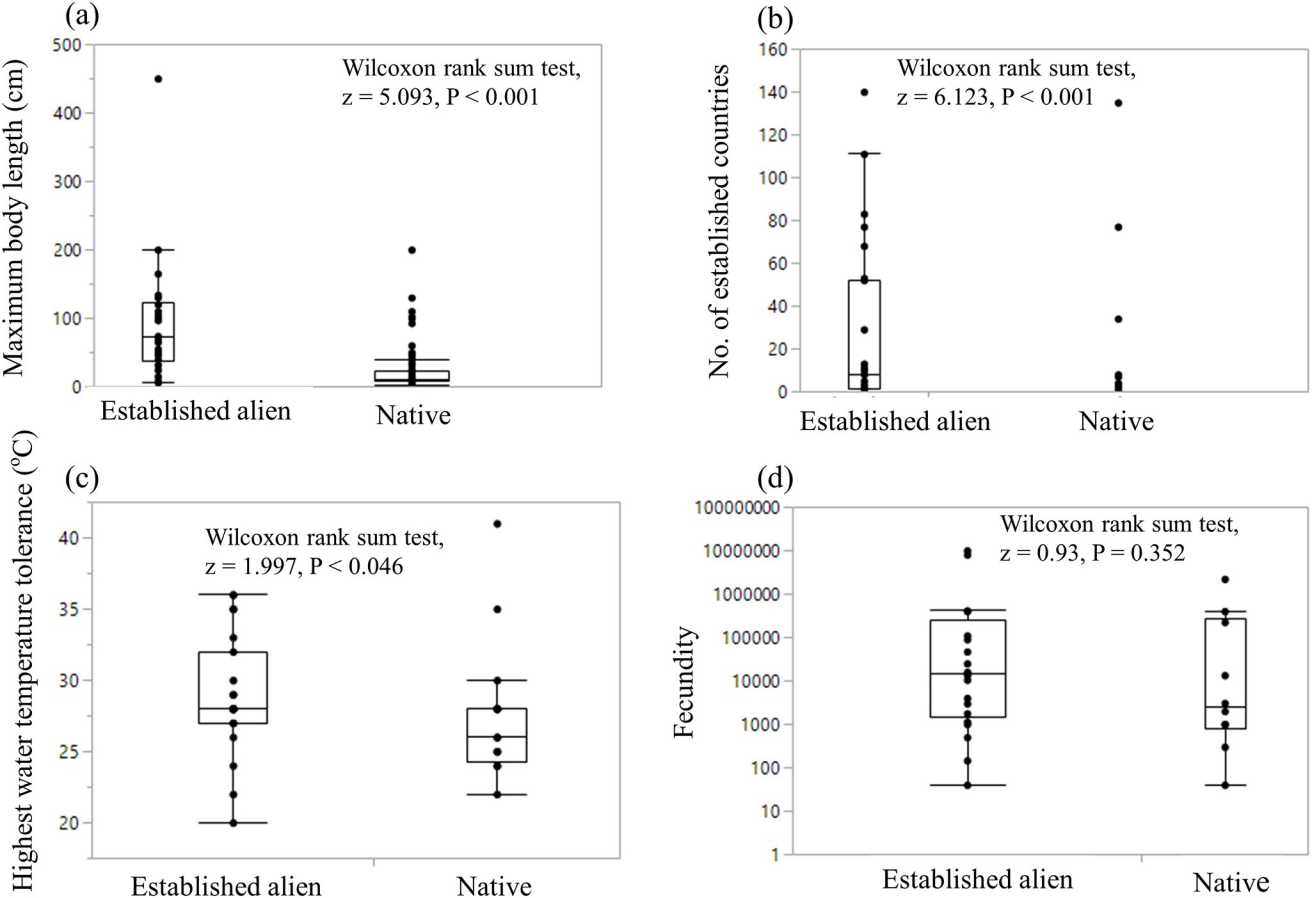
Box-plots comparing established alien species and native species for the four interval variables: (a) maximum body length (Wilcoxon rank sum test, z = 5.093, P < 0.001), (b) the number of established countries where the species was alien but has since established itself (Wilcoxon rank sum test, z = 6.123, P < 0.001), (c) the highest water temperature tolerance (°C) (Wilcoxon rank sum test, z = 1.997, P < 0.046), and (d) fecundity (Wilcoxon rank sum test, z = 0.93, P = 0.352). Sample sizes vary because data were not available for all species (see S1 and S2 Tables for species lists and associated variable information).

The five top-ranked variables from the final establishment success model (see above) were: AQUAC, MAXL, ESTAB, FECUN, and TEMPH. We tested each variable in turn to compare established alien species versus native species. Significantly more established alien species had aquaculture use than native species (χ_1_ = 30.537, *P* < 0.001). The maximum body lengths of the established alien species (91.24 ± 17.51 cm) were significantly longer than those of the native species (22.99 ± 3.76 cm) (Wilcoxon rank sum test, z = 5.093, P < 0.001) (Fig 3a). The established alien species had invaded significantly more countries than the native species (Wilcoxon rank sum test, z = 6.123, P < 0.001) (Fig 3b). The established alien species had significantly greater TEMPH than the TEMPH of the native species (Wilcoxon rank sum test, z = 1.997, P < 0.046) (Fig 3c). However, the fecundity was not significantly different between the established alien species and the native species (Wilcoxon rank sum test, z = 0.93, P = 0.352) (Fig 3d).

## Discussion

### Discussion of the results of our DT model and comparisons with native species

When we compared established versus non-established alien species, the DT methods showed that the five most important determinants of establishment success were aquaculture use, maximum body length, the number of established countries, fecundity, and the highest water temperature tolerance. Furthermore, the first four important determinants (aquaculture use, maximum body length, the number of established countries, fecundity) were also significantly different in our comparison between the randomly selected non-established vs. the established species. When we compared established alien species versus native species, we used those five determinants to investigate a life history hypothesis predicting differences between the two groups, and we found that all determinants except fecundity were associated with significantly higher levels in the established alien species than in the native species.

In our study, the establishment success was identified from the import stage to the establishment stage, and we did not differentiate the introduction stage from the establishment stage because there are no reliable records of introduction events in Taiwan (see Introduction). However, from the results of our variable exploration, aquaculture use, maximum body length, the number of established countries and fecundity were significantly different in both comparisons 1 and 2 (Table 2). That is, those variables might affect not only the outcome of the predefined invasion process, including both introduction stage and establishment stage, for imported species but also the outcome of the establishment stage for introduced species.

However, although a non-established alien species could fail at the introduction stage or establishment stage as the introduced non-established did, an alien species has to succeed at both the introduction stage and the establishment stage to become an established species in the freshwater habitats of Taiwan. Aquaculture use was the most important determinant of establishment success of alien freshwater fishes, and those established alien species had more aquaculture use than Taiwan’s native species. Therefore, our aquaculture variable might involve human use in affecting both the introduction and the establishment stage. Previous studies have shown that aquaculture activities are the main driver leading to introduction of alien freshwater fishes [40, 41]. We speculate that one of the reasons why aquaculture species are more likely to be introduced successfully in Taiwan is the regular flooding events which often happen during typhoons and other extreme weather events. This was suggested as a key factor for the introduction of alien fish species due to aquaculture activities in southern Brazil [41]. Consequently, we emphasize two management approaches suggested by previous studies: (1) an adequate biosecurity management for aquaculture farm facilities such as increasing pond embankment height or constructing containment structures to prevent escape events during flooding [41], and (2) a reduction in freshwater aquaculture [42]. Given the co-occurrences of increased aquaculture [43] and increasing flooding risks in Asia [44, 45] in recent years, there two management suggestions should also be considered for other Asian countries with extensive freshwater aquaculture.

A species used in aquaculture might be selected because of such traits as fast growth rate, high disease resistance, or earlier age at maturation [46], or because of its high adaptability to environmental stresses [47]. Thus, our finding that established alien species had significantly more aquaculture use than the native species might also suggest that species which were used in aquaculture and then escaped and became established in the wild are selected by fish breeders because of traits such as fast growth rate, high disease resistance, earlier age at maturation, or high adaptability to environmental stresses. Those alien aquaculture species with those traits might not only establish themselves successfully but also create unpredictable and irreversible ecological impacts [40, 48]. Therefore, the use of alien species in aquaculture is clearly a risk factor for establishment in Taiwan, as it is globally [3, 49].

The second most important variable of successful establishment was maximum body length. Species traits related to body size have been demonstrated to be the most important predictors in other fish invasion studies. For example, Kolar and Lodge [16] found that relative body length growth was the most important predictor which appeared at the top of their classification tree in predicting the successful establishment of alien fishes in the Great Lakes. Using a global dataset of 1424 freshwater fish introductions, Ruesink [17] showed that body size was the most important predictor which appeared at the top of her DT.

In Taiwan, imported alien fish species of greater body length had a higher establishment success, whether in aquaculture use or not (see third level in Fig 2, MAXL ≥ 37 cm for aquaculture use and MAXL ≥ 39.35 cm for no aquaculture use). Our results thus differ from Ruesink’s [17] results because she found that families with smaller body sizes had higher establishment probabilities. We speculate that these contrasting results may result from the differential species composition of the two data sets. In particular, our dataset of fish species without aquaculture use is composed primarily of ornamental fishes because they are very popular as pets in Taiwan. When well fed, ornamental fishes generally grow faster and larger than their wild congeners; in addition, fish owners probably release their fish into garden and park ponds, lakes, and rivers, once they get tired of them or their body length approaches or even exceeds the size of the aquarium or holding tank. Therefore, larger ornamental fish species may have a higher probability of being released and then successfully establishing themselves in Taiwan. Since Taiwan has a long history of aquaculture, which has recently expanded from food fish culture further into ornamental fish culture [50], the risk of alien species establishment likely to increase. The finding that alien fish species with a larger body length have a higher probability of successful establishment in Taiwan may also have affected the body length distributions of Taiwan’s freshwater fish assemblages. For example, the body length patterns may have shifted away from its original latitudinal patterns and disrupted the native ecosystem properties, as Blanchet et al. [51] suggested. When we compared the maximum body lengths of established alien species and native species, we found that established alien species had significantly longer body lengths. This finding is concordant with a global study in which invasive species were also larger in body size [52]. In fish, bigger is better in terms of survival [53]. Thus, our result supports a life history hypothesis that established alien freshwater fishes have better traits in body size than the native species.

The probability of failed establishment was 92.71% for fish species without aquaculture use, but the probability of failed establishment increased to 100% if four more conditions were met; specifically, the number of established countries and three species traits (the maximum body length, the fecundity, and the highest water temperature tolerance) (Fig 3). Furthermore, in comparing the number of established countries between established alien species and native species, we found that established alien species had invaded significantly more countries than the native species of Taiwan. This finding demonstrated that established alien species might have higher abilities which enable them to invade more diverse environments/countries than those of native species. Liang et al. [25] found a similar result for bird species and emphasized that future invasion success could be gauged by simply looking at previous success in invading other countries or regions. In other words, if a species has successfully invaded other countries before, it will probably be successful again.

Higher abilities with better traits may also be reflected in our finding that established alien species have a significantly greater highest water temperature tolerance than that of the native species. Given that climate change is inexorably increasing temperatures in Taiwan [54], this tolerance advantage is likely to become even more important. However, even though the highest water temperature tolerance was an important determinant for the establishment success of imported alien species, the insignificant results of our comparison between the randomly selected non-established (assumed as introduced non-established) vs. established alien species suggested that once the imported species passed the introduction stage and became an introduced species, the highest water temperature tolerance might not have a significant effect on the outcome of the establishment stage for those introduced species.

Finally, we found that the fecundity measured as the maximum number of eggs was not significantly different between the established alien and native species. Therefore, except for their reproductive potential, our results supported a life history hypothesis in that established alien species have different (presumably better) traits than the native species as shown in their higher use in aquaculture, larger body sizes, the number of established countries, and tolerance of greater highest water temperatures.

### Performance and limitations of our DT model

When we compared the established alien species to the non-established alien species, our results showed that the gradient boosting method performed better than the other three DT methods based on the total scores, and this method also outperformed the other methods for three out of the five performance measures, namely, precision, recall, and accuracy. This finding mirrors that of our previous study [25] of establishment success of exotic birds in Taiwan in which the gradient boosting method also performed best based on the total scores achieved by the same four DT methods.

However, our results were somewhat different from the fish DT models published by Keller et al. [19] who calculated only two performance measures, namely AUC and accuracy. First, our best performing model was the gradient boosting model, but in Keller et al. [19] it was not the best except in one case. Keller et al. [19] examined two separate fish datasets. The gradient boosting model only performed the best for the Great Lakes fish data set (45 species with 17 predictor variables) when considering the AUC measure; when considering the accuracy measure, it was the second best model. For the California fish dataset (87 species with 7 predictor variables), the gradient boosting model was the worst performing one among the three DT models when considering the AUC measure; when considering the accuracy measure, it was the second best performing one. Second, our gradient boosting models had AUC (0.903–0.913) and accuracy (0.932) values higher than those (AUC: 0.662–0.871; accuracy: 0.540–0.778) achieved by the fish DT models of Keller et al. [19]. Thus, our high AUC values (> 0.9) indicate a very good discrimination ability of our models according to Pearce and Ferrier [55]. Our overall good discrimination ability and the high accuracy of our gradient boosting models may have resulted from the inclusion of more predictor variables (up to 17 predictor variables) rather than the sample size [19, 25].

When we compared the performance of variable treatment I with that of variable treatment II (excluding taxon variables), we found very little difference in model performance, again mirroring the results of Liang et al. [25]. Although the ORDER and FAMILY variables were excluded in treatment II, their exclusion did not affect the model performance much. However, the importance of the order variable when it was included in our establishment success model suggests that it nevertheless may be an important variable and should be included as a reference either in future management or hypothesis testing studies [13].

Although we performed DT methods to test the two proposed hypotheses in freshwater fish invasion in Taiwan, there are some limitations in our study. First, we only considered the species characteristics and its associated factors and did not take into account the interspecific interaction factors such as facilitation, as suggested by the invasion meltdown hypothesis, which was first proposed by Simberloff and Holle [56] and was then supported by the study of species invading the Great Lakes [57]. Furthermore, abiotic factors such as water temperature and biotic factors such as invasive fish may have combinatory or even synergistic effects on native fish. For example, water temperature not only separated fish species distribution but also combined with alien fish invasions affected the distribution of native species [58]. Therefore, further studies on the 26 established alien species identified in this study and their impacts on native species should take these possible interactions and combinatory effects into consideration.

Another limitation of our study is the availability of data sources. For some data fields, we had missing information, e.g., FECUN or TEMPH for native species had 87% and 74% of missing data fields, respectively. Therefore, to improve analyses, we also need to conduct more studies which investigate basic biological information about Taiwan’s native species.

In summary, we conclude that the gradient boosting models performed best for predicting the establishment success of imported alien freshwater fishes in Taiwan. Our results demonstrate that the two most important determinants for predicting establishment success in Taiwan are aquaculture use and maximum body length. Because of our results, we suggest that management priorities should be focused on alien fish species which are used in aquaculture and which have larger body sizes, certainly in Taiwan and possibly also in other countries where conditions are similar to Taiwan. Finally, our study supported a life history hypothesis which states that established alien freshwater fishes have better abilities (reflected, e.g., in higher aquaculture use and a higher number of previously invaded countries) and also different traits in body size and tolerance of the highest water temperature when compared to the native freshwater fishes of Taiwan.

We suggest that the species characteristics associated with establishment success may alert researchers, conservation managers and decision-makers to species with a high establishment potential, especially for countries with similar conditions as those in Taiwan. Many tropical and subtropical countries in East Asia have similar conditions to Taiwan (rapidly expanding economies which allow people money and leisure time to pursue activities such as keeping of ornamental fish and prayer release, more money spent on protein-rich foods including fish, rather lax laws about alien species, etc.). We suggest that our results are especially relevant to East Asian counties, but also other countries with similar conditions as Taiwan.

## Supporting information

S1 Table List of 118 alien freshwater fish species which were imported into Taiwan and their associated variable information (see Table 1 for variable descriptions). (PDF)

S2 Table List of 77 native freshwater fish species and their associated variable information (see Table 1 for variable descriptions). (PDF)
