# Supplementary material for: Determinants of establishment success: Comparing alien and native freshwater fishes in Taiwan
Source: PLoS One. 2020 Jul 23;15(7):e0236427. doi: 10.1371/journal.pone.0236427 (PMC7377439; doi:10.1371/journal.pone.0236427)
Supplement: S1 Table — (PDF) [file pone.0236427.s001.pdf]

Table S1. List of 118 alien freshwater fish species which were imported into Taiwan and their associated variable information (see Table 1 for variable descriptions).

SPECIES	ORDER	FAMILY	LOVERLAP	DIET	AQUAC	PET	PHL	PHH	DHL	DHH	TEMPL	TEMPH	MAXL	INTROD	ESTAB	FOODIT	FECUN	SUCCESS
Acipenser sinensis	Acipenseriformes	Acipenseridae	2	4	1	1	.	.	.	.	15	20	130.0	1	0	3	1303000	0
Aequidens rivulatus	Perciformes	Cichlidae	0	4	2	2	6.5	8.0	5	13	20	24	20.0	1	0	4	600	0
Altolamprologus compressiceps	Perciformes	Cichlidae	0	4	1	1	6.5	7.5	8	12	23	25	12.3	1	0	2	300	0
Amphilophus citrinellus	Perciformes	Cichlidae	0	3	1	2	6.6	7.3	10	20	23	33	24.4	6	5	6	1000	1
Amatitlania nigrofasciata	Perciformes	Cichlidae	0	3	1	2	7.0	8.0	9	20	20	36	10.0	11	9	7	150	0
Anguilla australis	Anguilliformes	Anguillidae	0	3	2	2	.	.	.	.	.	.	130.0	4	0	4	10000000	1
Ancistrus hoplogenys	Siluriformes	Loricariidae	0	2	1	1	6.0	7.0	5	12	25	28	15.8	1	0	1	.	0
Anguilla reinhardtii	Anguilliformes	Anguillidae	0	4	2	2	.	.	.	.	.	.	165.0	1	0	4	10000000	1
Apistogramma agassizii	Perciformes	Cichlidae	0	4	1	2	5.0	7.0	2	10	26	29	4.2	1	0	3	150	0
Apistogramma bitaeniata	Perciformes	Cichlidae	0	4	1	2	5.0	6.0	2	6	23	25	4.6	1	0	2	60	0
Apistogramma borelli	Perciformes	Cichlidae	0	4	1	2	6.0	8.0	5	19	24	25	3.9	1	0	4	70	0
Apistogramma cacatuoides	Perciformes	Cichlidae	0	4	1	2	6.0	8.0	5	19	24	25	5.0	1	0	2	80	0
Apistogramma eunotus	Perciformes	Cichlidae	0	4	1	2	7.0	7.5	8	10	23	30	5.3	1	0	2	150	0
Apistogramma hoignei	Perciformes	Cichlidae	0	3	1	1	5.5	7.0	1	15	23	29	6.0	1	0	2	.	0
Apistogramma hongsloi	Perciformes	Cichlidae	0	4	1	1	5.5	6.0	5	10	23	30	5.0	1	0		90	0
Apistogramma iniridae	Perciformes	Cichlidae	0	4	1	1	5.5	6.0	4	18	23	31	5.0	1	0	2	120	0
Apistogramma nijsseni	Perciformes	Cichlidae	0	4	1	2	5.0	5.6	1	5	23	30	3.9	1	0	3	.	0
Apistogramma norberti	Perciformes	Cichlidae	0	4	1	1	5.8	6.8	0	4	23	28	3.9	1	0	2	.	0

Apistogramma pertensis	Perciformes	Cichlidae	0		1	2	5.5	.	5	.	23	30	4.5	1	0		120	0
Apistogrammoides pucallpaensis	Perciformes	Cichlidae	0	4	1	1	6.0	7.0	0	5	23	30	2.7	1	0	2	100	0
Apistogramma trifasciata	Perciformes	Cichlidae	0	4	1	2	6.0	6.5	2	5	26	29	3.8	1	0	2	100	0
Apistogramma viejita	Perciformes	Cichlidae	0	3	1	2	5.5	6.5	1	5	23	30	2.7	1	0	2	100	0
Arapaima gigas	Osteoglossiformes	Arapaimidae	0	4	2	2	6.0	6.5	5	12	25	29	450.0	8	2	1	47000	1
Archocentrus spilurus / Cryptoheros spilurus	Perciformes	Cichlidae	0	3	1	2	6.0	7.5	5	10	22	32	12.0	1	1	2	400	0
Astronotus ocellatus	Perciformes	Cichlidae	0	3	1	2	6.0	8.0	5	19	22	25	45.7	14	6	7	2000	0
Aulonocara baenschi	Perciformes	Cichlidae	0	4	1	2	7.2	8.2	10	30	22	26	13.0	1	0	1	.	0
Aulonocara jacobfreibergi	Perciformes	Cichlidae	0	4	1	2	7.5	8.0	9	19	24	26	15.0	1	0	3	50	0
Aulonocara stuartgranti	Perciformes	Cichlidae	0	4	1	2	7.7	8.6	6	10	22	26	11.8	1	0	1	.	0
Barbonymus schwanenfeldii	Cypriniformes	Cyprinidae	0	3	2	2	6.5	7.0	2	10	22	25	35.0	6	2	7	16000	0
Betta splendens	Perciformes	Osphronemidae	0	4	1	2	6.0	8.0	5	19	24	30	6.5	10	6	3	390	0
Botia macracanthus / Chromobotia macracanthus	Cypriniformes	Cobitidae	0	3	1	2	5.0	8.0	5	12	25	30	30.5	4	0	4	.	0
Carassius auratus	Cypriniformes	Cyprinidae	2	3	2	2	6.0	8.0	5	19	18	22	32.0	88	77	7	400000	1
Channa micropeltes	Perciformes	Channidae	0	4	2	2	6.0	8.0	8	12	25	28	130.0	4	1	5	.	1
Chitala ornata	Osteoglossiformes	Notopteridae	0	4	1	2	5.5	7.0	2	10	24	28	100.0	4	3	4	.	1
Channa striata	Perciformes	Channidae	2	4	2	2	7.0	8.0	.	20	23	27	100.0	13	8	8	15989	1
Cichlasoma bifasciatum / Paraneetroplus bifasciatus	Perciformes	Cichlidae	0	3	1	1	6.5	7.5	2	15	26	30	30.0	1	0	2	500	0

Cichlasoma managuense	Perciformes	Cichlidae	2	4	2	2	7.0	8.7	10	15	25	36	55.0	15	13	3	10496	1
Cichla ocellaris	Perciformes	Cichlidae	2	4	2	2	6.5	7.5	5	12	24	27	74.0	10	10	1	15000	1
Cichlasoma salvini	Perciformes	Cichlidae	0	4	1	2	7.0	8.0	5	20	22	32	22.0	3	1	4	600	0
Clarias batrachus	Siluriformes	Clariidae	2	3	2	2	5.5	8.0	2	25	10	28	47.0	11	10	9	13400	1
Copadichromis borleyi	Perciformes	Cichlidae	0	4	1	2	8.0	8.5	10	25	24	26	14.0	1	0	1	60	0
Colisa fasciatus / Trichogaster fasciata	Perciformes	Osphronemidae	0	3	1	1	6.0	7.5	4	15	22	28	12.5	4	3		600	0
Colisa labiosus / Trichogaster labiosa	Perciformes	Osphronemidae	0	4	1	2	6.0	7.5	4	10	22	28	9.0	4	3	2	600	0
Ctenopoma acutirostre	Perciformes	Anabantidae	0	4	1	2	6.0	8.0	6	20	20	25	15.0	1	0	3	.	0
Cynotilapia afra	Perciformes	Cichlidae	0	4	1	2	8.0	8.5	10	25	23	27	10.1	1	0	1	.	0
Cyprinus carpio	Cypriniformes	Cyprinidae	0	3	2	2	7.0	7.5	10	15	3	35	110.0	173	140	9	416650	1
Cyrtocara compressiceps / Dimidiochromis compressiceps	Perciformes	Cichlidae	0	4	1	2	8.0	8.0	9	19	22	28	23.0	1	0	1	.	0
Cyphotilapia frontosa	Perciformes	Cichlidae	0	4	1	2	7.8	9.0	8	12	24	26	33.0	1	0	4	50	0
Cyathopharynx furcifer	Perciformes	Cichlidae	0	4	1	2	8.0	9.0	9	19	24	26	21.0	1	0	1	40	0
Cyprichromis leptosoma	Perciformes	Cichlidae	0	4	1	2	8.0	9.0	9	19	23	25	11.0	1	0	4	.	0
Cyprichromis leptosoma	Perciformes	Cichlidae	0	3	1	2	8.0	9.0	9	19	23	25	11.0	1	0	4	.	0
Cyrtocara moorii	Perciformes	Cichlidae	0	4	1	2	7.2	8.8	10	18	24	26	20.0	1	0	2	90	0
Dicrossus filamentosus	Perciformes	Cichlidae	0	3	1	2	5.0	7.0	5	8	23	25	3.8	1	0	2	120	0
Dicrossus maculatus	Perciformes	Cichlidae	0	3	1	1	4.8	6.0	1	5	22	25	6.0	1	0	3	.	0
Fossorochromis rostratus	Perciformes	Cichlidae	0	4	1	2	7.2	8.8	10	18	24	26	24.4	1	0	3	.	0

Gambusia affinis	Cyprinodontiformes	Poeciliidae	0	4	1	2	6.0	8.0	5	19	12	29	7.0	82	68	6	146	1
Geophagus surinamensis	Perciformes	Cichlidae	0	3	1	2	6.0	8.0	5	19	22	25	14.8	3	2	3	250	0
Gymnogeophagus balzanii	Perciformes	Cichlidae	0	4	1	2	6.0	8.0	5	19	22	26	12.0	1	0	3	500	0
Hemichromis cristatus / Hemichromis bimaculatus	Perciformes	Cichlidae	0	4	1	1	6.5	7.5	4	16	21	23	13.6	5	4	3	799	0
Heros severus	Perciformes	Cichlidae	0	3	1	2	5.0	6.5	4	6	23	29	20.0	2	0	7	200	0
Hyphessobrycon amandae	Characiformes	Characidae	0	4	1	2	6.5	7.0	6	10	24	28	2.0	1	0	2	.	0
Hyphessobrycon anisitsi	Characiformes	Characidae	0	3	1	2	6.0	9.0	2	30	18	28	6.0	1	0	5	.	0
Hyphessobrycon bentosi	Characiformes	Characidae	0	3	1	2	5.8	7.5	5	19	24	28	4.3	1	0	2	.	0
Hyphessobrycon callistus / Hyphessobrycon eques	Characiformes	Characidae	0	3	1	2	5.0	7.8	10	25	22	26	4.0	5	1	7	.	0
Hyphessobrycon ecuasoriensis / Hyphessobrycon columbianus	Characiformes	Characidae	0	3	1	2	5.0	7.0	3	12	23	28	7.0	1	0	5	.	0
Hyphessobrycon erythrostigma	Characiformes	Characidae	0	3	1	2	5.6	7.2	8	12	23	28	6.1	3	0	4	.	0
Hyphessobrycon flammeus	Characiformes	Characidae	0	3	1	2	5.8	7.8	5	25	22	28	2.5	1	0	4	300	0
Hypostomus plecostomus	Siluriformes	Loricariidae	0	3	1	2	6.2	8.2	1	25	20	28	50.0	13	8	4	3000	1
Hyphessobrycon pulchripinnis	Characiformes	Characidae	0	3	1	2	5.5	8.0	3	20	23	28	3.8	3	0	5	.	0
Hyphessobrycon scholzei	Characiformes	Characidae	0	3	1	2	6.8	8.0	.	25	22	28	4.0	1	0	5	.	0
Hypselecara temporalis	Perciformes	Cichlidae	0	3	1	2	5.0	7.5	0.3	20	25	30	22.0	1	0	4	.	0
Hyphessobrycon vilmae	Characiformes	Characidae	0	3	1	2	5.0	7.0	1		22	26	4.0	1	0	2	.	0
Inpaichthys kerri	Characiformes	Characidae	0	4	1	2	6.0	8.0	5	12	24	27	2.8	1	0	2	.	0
Julidochromis dickfeldi	Perciformes	Cichlidae	0	3	1	2	8.5	9.2	8	14	22	25	11.0	1	0	2	.	0

Labidochromis caeruleus	Perciformes	Cichlidae	0	3	1	2	7.2	8.8	10	20	23	26	8.1	1	0	4	30	0
Labeotropbeus fuelleborni	Perciformes	Cichlidae	0	3	1	2	7.5	8.5	10	12	22	25	8.7	1	1	7	.	0
Megalobrama amblycephala	Cypriniformes	Cyprinidae	1	2	2	1	.	.	.		10	20	200.0	4	3	1	.	1
Melanochromis auratus	Perciformes	Cichlidae	0	2	1	2	7.0	8.5	10	15	22	26	11.0	1	0	1	40	0
Mesonauta festiva / Mesonauta festivus	Perciformes	Cichlidae	0	3	1	2	6.0	8.0	4	6	25	34	8.2	1	0	7	500	0
Megalamphodus megalopterus	Characiformes	Characidae	0	4	1	2	6.0	7.5	2	20	22	28	3.6	1	0	5	.	0
Megalamphodus sweglesi	Characiformes	Characidae	0	4	1	2	5.5	7.5	1	12	20	23	3.2	1	0	3	.	0
Micropterus salmoides	Perciformes	Centrarchidae	0	4	2	2	7.0	7.5	10	.	10	32	97.0	82	53	11	25000	1
Nannacara anomala	Perciformes	Cichlidae	0	4	1	2	6.0	8.0	5	19	22	25	5.6	1	0	4	300	0
Nanochromis parilus	Perciformes	Cichlidae	0	4	1	2	6.0	7.0	5	8	22	25	4.4	1	0	4	250	0
Neolamprologus brichardi	Perciformes	Cichlidae	0	3	1	1	8.0	9.0	9	19	22	25	9.0	1	0	1	200	0
Neolamprologus caudopunctatus	Perciformes	Cichlidae	0	1	1	2	7.5	8.5	15	.	23	25	6.5	1	0	1	.	0
Neolamprologus fasiantus / Neolamprologus fasciatus	Perciformes	Cichlidae	0	4	1	1	8.5	9.0	24	26	23	25	15.0	1	0	.	.	0
Neolamprologus leleupi	Perciformes	Cichlidae	0	4	1	2	7.5	8.0	12	15	24	26	10.0	1	0	1	150	0
Nimbochromis venustus	Perciformes	Cichlidae	0	4	1	1	7.2	8.8	10	18	25	27	25.0	1	0	1	120	0
Oncorhynchus mykiss	Salmoniformes	Salmonidae	0	4	2	1	6.5	8.5	.	.	10	24	120.0	127	83	10	4000	1
Ophthalmotilapia ventralis	Perciformes	Cichlidae	0	3	1	1	7.5	8.0	10	12	23	25	15.0	1	0	4	60	0
Oreochromis mossambicus	Perciformes	Cichlidae	0	3	2	2	.	.	.	.	17	35	39.0	128	111	11	1775	1
Oxyeleotris marmorata	Perciformes	Eleotridae	1	3	2	2	6.5	7.5	10	15	22	28	65.0	3	1	5	90000	1

Paracheirodon Innesi	Characiformes	Characidae	0	3	1	2	5.0	7.0	1	2	20	26	2.2	5	1	4	130	0
Papiliochromis milomo	Perciformes	Cichlidae	0	4	1	2	8.0	8.5	10	20	23	26	18.7	1	0	3	.	0
Parambassis ranga	Perciformes	Ambassidae	2	4	1	2	7.0	8.0	9	19	20	30	8.0	2	1	3	500	1
Papiliochromis ramirezi	Perciformes	Cichlidae	0	3	1	2	5.0	6.0	5	12	27	30	3.4	1	0	3	200	0
Pelvicachromis pulcher	Perciformes	Cichlidae	0	4	1	1	5.0	8.0	5	19	24	25	11.0	3	1	4	300	0
Phractocephalus hemioliopterus	Siluriformes	Pimelodidae	0	3	2	2	5.5	6.8	9	10	20	26	134.0	2	0	4	.	1
Plecoglossus altivelis	Osmeriformes	Plecoglossidae	1	3	2	2	.	.	.	.		.	70.0	3	1	7	110000	1
Poecilia reticulata	Cyprinodontiformes	Poeciliidae	0	4	1	1	7.0	8.0	9	19	18	28	6.0	62	52	2	40	1
Pseudotropheus elongatus	Perciformes	Cichlidae	0	2	1	2	7.5	8.0	9	19	22	25	9.5	1	0	1	37	0
Pseudoplatystoma fasciatum	Siluriformes	Pimelodidae	0	4	1	2	6.0	8.0	4	30	24	28	104.0	1	0	3	8000000	1
Pseudotropheus zebra	Perciformes	Cichlidae	0	3	1	1	8.0	8.0	9	19	22	28	11.3	1	0	2	60	0
Pterophyllum altum	Perciformes	Cichlidae	0	3	1	1	4.8	6.2	1	5	27	31	18.0	1	0	3	.	0
Pterophyllum scalare	Perciformes	Cichlidae	0	4	1	2	6.0	8.0	5	13	24	30	7.5	7	1	3	.	0
Puntius titteya	Cypriniformes	Cyprinidae	0	3	1	2	6.0	8.0	5	19	23	27	5.0	3	2	3	300	0
Rasbora borapetensis	Cypriniformes	Cyprinidae	0	4	1	2	6.5	7.0	2	12	22	26	6.0	2	1	5	40	0
Symphysodon aequifasciatus	Perciformes	Cichlidae	0	4	1	2	5.0	8.0	6	13	26	30	13.7	3	0	5	.	0
Tanichthys albonubes	Cypriniformes	Cyprinidae	1	4	1	2	6.0	8.0	5	19	18	22	4.0	6	3	2	300	0
Thorichthys meeki	Perciformes	Cichlidae	0	3	1	2	6.5	7.5	8	15	26	30	17.0	7	5	4	500	0
Tilapia zillii	Perciformes	Cichlidae	2	3	2	2	6.0	9.0	5	20	11	36	40.0	37	29	8	1147	1
Trichogaster lalia / Trichogaster lalius	Perciformes	Osphronemidae	0	3	1	2	6.0	8.0	5	19	25	28	8.8	6	3	2	600	0
Trichogaster leeri	Perciformes	Osphronemidae	0	3	2	2	6.0	8.0	5	19	24	28	12.0	5	3		300	0

Trichogaster microlepis	Perciformes	Osphronemidae	0	4	1	2	6.0	7.0	2	25	26	30	13.0	5	3	3	2000	0
Trichogaster trichopterus	Perciformes	Osphronemidae	0	4	2	2	6.0	8.0	5	19	22	28	15.0	13	11	4	.	1
Xiphophorus helleri	Cyprinodontiformes	Poeciliidae	2	3	1	2	7.0	8.0	9	19	22	28	16.0	32	28	5	.	0
Xiphophorus maculatus	Cyprinodontiformes	Poeciliidae	0	4	1	2	7.0	8.0	9	19	18	25	6.0	21	17	2	.	0

"." = missing value.
