# Supplementary material for: Determinants of establishment success: Comparing alien and native freshwater fishes in Taiwan
Source: PLoS One. 2020 Jul 23;15(7):e0236427. doi: 10.1371/journal.pone.0236427 (PMC7377439; doi:10.1371/journal.pone.0236427)
Supplement: S2 Table — (PDF) [file pone.0236427.s002.pdf]

Table S2. List of 77 native freshwater fish species and their associated variable information (see Table 1 for variable descriptions).

Species	Order	Family	AQUAC	TEMPH	MAXL	ESTAB	FECUN
Anguilla marmorata	Anguilliformes	Anguillidae	y	.	200.0	0.0	.
Oryzias latipes	Beloniformes	Adrianichthyidae	n	24	4.0	8.0	40.0
Formosania lacustre	Cypriniformes	Balitoridae	n	.	15.0	0.0	.
Sinogastromyzon nantaiensis	Cypriniformes	Balitoridae	n	.	8.0	0.0	.
Sinogastromyzon puliensis	Cypriniformes	Balitoridae	n	30	9.0	0.0	.
Hemimyzon formosanus	Cypriniformes	Balitoridae	n	.	10.0	0.0	.
Hemimyzon sheni	Cypriniformes	Balitoridae	n	.	5.0	0.0	.
Hemimyzon taitungensis	Cypriniformes	Balitoridae	n	.	12.0	0.0	.
Cobitis cf. sinensis	Cypriniformes	Cobitidae	n	.	13.5	0.0	.
Cobitis sp.	Cypriniformes	Cobitidae	y	.	10.0	0.0	.
Misgurnus anguillicaudatus	Cypriniformes	Cobitidae	y	25	10.0	0.0	2000.0
Paramisgurnus dabryanus	Cypriniformes	Cobitidae	y	25	9.0	1.0	.
Acrossocheilus paradoxus	Cypriniformes	Cyprinidae	y	25	22.5	0.0	.
Aphyocypris kikuchii	Cypriniformes	Cyprinidae	n	.	8.0	0.0	.
Candidia barbatus	Cypriniformes	Cyprinidae	n	.	20.0	0.0	.
Candidia pingtungensis	Cypriniformes	Cyprinidae	n	.	11.4	0.0	.
Carassius auratus	Cypriniformes	Cyprinidae	y	41	32.0	77.0	400000.0
Chanodichthys erythropterus	Cypriniformes	Cyprinidae	y	.	102.0	0.0	.
Culter alburnus	Cypriniformes	Cyprinidae	n	.	92.8	0.0	.
Cyprinus carpio	Cypriniformes	Cyprinidae	y	35	110.0	135.0	2208000.0

Distoechodon tumirostris	Cypriniformes	Cyprinidae	n	.	35.0	0.0	.
Gobiobotia cheni	Cypriniformes	Cyprinidae	n	.	10.0	0.0	.
Gobiobotia kolleri	Cypriniformes	Cyprinidae	n	.	12.0	0.0	.
Hemibarbus labeo	Cypriniformes	Cyprinidae	n	.	40.0	1.0	.
Hemiculter leucisculus	Cypriniformes	Cyprinidae	n	22	25.0	7.0	.
Metzia formosae	Cypriniformes	Cyprinidae	n	.	12.0	0.0	.
Metzia mesembrinum	Cypriniformes	Cyprinidae	n	.	8.0	1.0	.
Microphysogobio alticorpus	Cypriniformes	Cyprinidae	n	.	7.0	0.0	.
Microphysogobio brevirostris	Cypriniformes	Cyprinidae	n	.	9.0	0.0	.
Onychostoma alticorpus	Cypriniformes	Cyprinidae	n	.	50.0	0.0	.
Onychostoma barbatulum	Cypriniformes	Cyprinidae	n	.	45.0	0.0	.
Opsariichthys evolans	Cypriniformes	Cyprinidae	n	.	12.0	0.0	.
Opsariichthys kaopingensis	Cypriniformes	Cyprinidae	n	.	11.4	0.0	.
Opsariichthys pachycephalus	Cypriniformes	Cyprinidae	n	.	16.0	0.0	.
Pararasbora moltriechti	Cypriniformes	Cyprinidae	n	.	8.0	0.0	.
Pseudorasbora parva	Cypriniformes	Cyprinidae	n	22	11.0	34.0	3060.0
Puntius semifasciolata	Cypriniformes	Cyprinidae	n	24	10.0	2.0	300.0
Puntius snyderi	Cypriniformes	Cyprinidae	n	.	9.0	0.0	.
Rhodeus ocellatus	Cypriniformes	Cyprinidae	n	24	6.0	4.0	.
Sinibrama macrops	Cypriniformes	Cyprinidae	n	.	22.7	0.0	.
Spinibarbus hollandi	Cypriniformes	Cyprinidae	n	.	60.0	0.0	.
Squalidus argentatus	Cypriniformes	Cyprinidae	n	.	13.0	0.0	.

Squalidus banarescui	Cypriniformes	Cyprinidae	n	.	10.0	0.0	.
Squalidus iijimae	Cypriniformes	Cyprinidae	n	.	10.0	0.0	.
Tanakia chii	Cypriniformes	Cyprinidae	n	28	9.0	0.0	.
Tanakia himantegus	Cypriniformes	Cyprinidae	n	28	8.0	0.0	.
Macropodus opercularis	Perciformes	Osphronemidae	n	26	6.7	2.0	.
Channa asiatica	Perciformes	Channidae	n	28	25.0	1.0	.
Channa maculata	Perciformes	Channidae	y	.	45.0	3.0	.
Awaous melanocephalus	Perciformes	Gobiidae	n	.	15.0	0.0	.
Awaous ocellaris	Perciformes	Gobiidae	n	.	15.5	0.0	.
Glossogobius celebius	Perciformes	Gobiidae	n	.	14.0	0.0	.
Lentipes armatus	Perciformes	Gobiidae	n	.	8.0	0.0	.
Mugilogobius myxodermus	Perciformes	Gobiidae	n	.	3.4	0.0	.
Oligolepis acutipennis	Perciformes	Gobiidae	n	26	15.0	0.0	.
Redigobius bikolanus	Perciformes	Gobiidae	n	28	4.9	0.0	1000.0
Rhinogobius candidianus	Perciformes	Gobiidae	n	.	7.8	0.0	.
Rhinogobius delicatus	Perciformes	Gobiidae	n	.	6.5	0.0	.
Rhinogobius formosanus	Perciformes	Gobiidae	n	.	4.9	0.0	.
Rhinogobius gigas	Perciformes	Gobiidae	n	.	8.1	0.0	.
Rhinogobius giurinus	Perciformes	Gobiidae	n	.	11.4	1.0	.
Rhinogobius henchuenensis	Perciformes	Gobiidae	n	.	9.0	0.0	.
Rhinogobius lanyuensis	Perciformes	Gobiidae	n	.	6.7	0.0	.
Rhinogobius maculafasciatus	Perciformes	Gobiidae	n	.	5.0	0.0	.

Rhinogobius nantaiensis	Perciformes	Gobiidae	n	.	6.6	0.0	.
Rhinogobius rubromaculatus	Perciformes	Gobiidae	n	.	3.4	0.0	.
Schismatogobius ampluvinculus	Perciformes	Gobiidae	n	.	2.7	0.0	.
Schismatogobius roxasi	Perciformes	Gobiidae	n	.	4.6	0.0	.
Sicyopterus japonicus	Perciformes	Gobiidae	n	.	15.0	0.0	224960.0
Stiphodon atropurpureus	Perciformes	Gobiidae	n	.	5.0	0.0	.
Stiphodon percnopterygionus	Perciformes	Gobiidae	n	.	3.7	0.0	.
Rhyacichthys aspro	Perciformes	Rhyacichthyidae	n	.	25.0	0.0	.
Tachysurus adiposalis	Siluriformes	Bagridae	n	.	22.3	0.0	.
Tachysurus brevianalis taiwanensis	Siluriformes	Bagridae	n	.	20.0	0.0	.
Clarias batrachus	Siluriformes	Clariidae	y	28	47.0	8.0	13400.0
Silurus asotus	Siluriformes	Siluridae	y	25	130.0	0.0	.
Monopterus albus	Synbranchiformes	Synbranchidae	y	28	100.0	2.0	1000.0

“.” = missing value.
